# Supplementary material for: Structural basis of specific H2A K13/K15 ubiquitination by RNF168
Source: Nat Commun. 2019 Apr 15;10:1751. doi: 10.1038/s41467-019-09756-z (PMC6465349; doi:10.1038/s41467-019-09756-z)
Supplement: Supplementary file 1 — Supplementary Information [file 41467_2019_9756_MOESM1_ESM.pdf]

Structural basis of specific H2A K13/K15 ubiquitination by RNF168

Horn, Uckelmann et al.

Supplementary Information

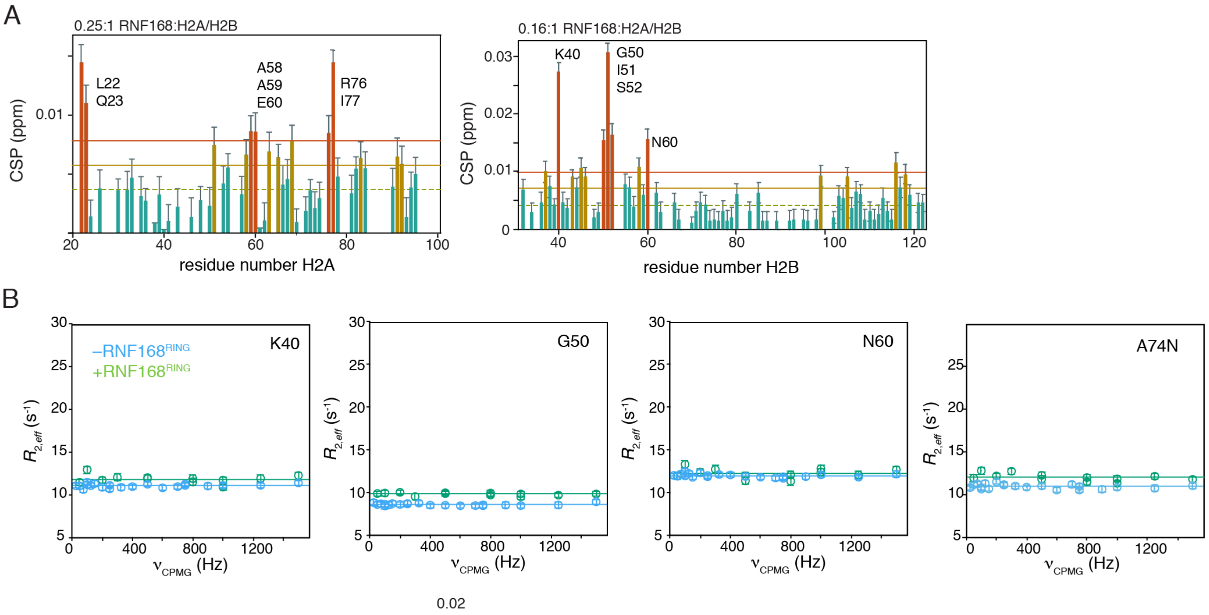

Supplementary Figure 1. Non-specific binding of RNF168^{RING} to the DNA binding regions of the H2A/H2B dimer. (A) Chemical shift perturbations (CSPs) for H2A (left) and H2B (right) within H2A/H2B dimers upon addition of sub-stoichiometric amounts of RNF168^{RING} (indicated in Figure). Residues with CSPs higher that are one (two) standard deviation (s.d.) higher than the one-sided 10% trimmed mean are color coded yellow (red) and labeled. (B) Addition of RNF168^{RING} does not induce dispersion of effective ^{15}N transverse relaxation rates, $R_{2,\mathrm{eff}}$ for H2B residues K40, G50 and N60 that are part of or close to the DNA binding regions of the H2A/H2B dimer. Residue A47 is not affected by addition of RNF168-RING and is shown as control. Exchange with the RNF168^{RING}-H2A-H2B complex causes a slight increase in $R_{2,\mathrm{eff}}$. Color coding indicated in Figure. Best-fit straight solid lines are shown to guide the eye. Error bars are s.d. based on 1/10 ppb errors in ^{1}H/^{15}N peak position in panel A and based on three replicate data points in panel B.

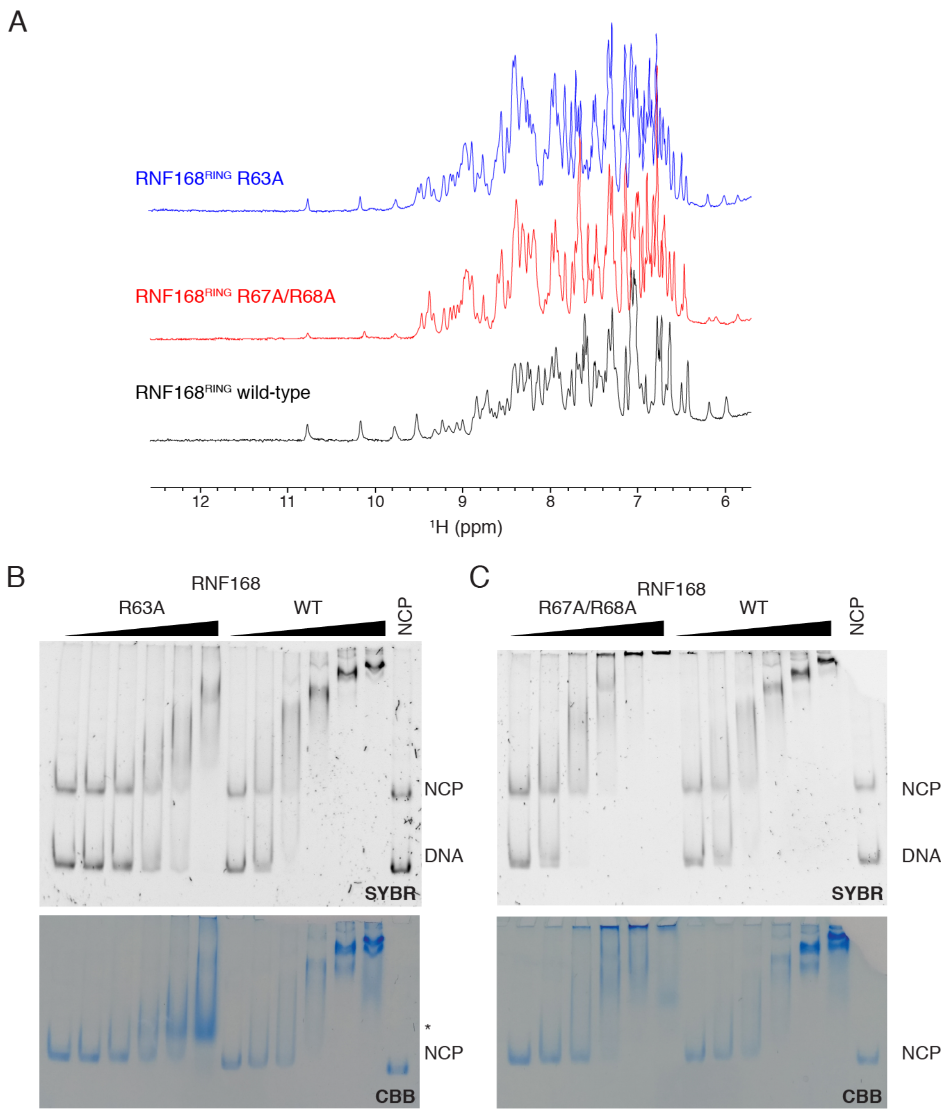

Supplementary Figure 2. RNF168RING R63A and R67/R68A mutants are folded and have altered nucleosome binding. (A) ^{1}H NMR spectra of wild-type, R63A, and R67/R68A mutant RNF168RING showing the amide-region. The high chemical shift dispersion and the similarity of the peak pattern between mutant and wild-type indicate that the mutants retain the native fold. (B,C) Native PAGE electrophoretic mobility shift assay (EMSA) of wild-type (WT)), R63A (B), and R67/R68A mutant (C) RNF168RING binding to nucleosomes; gel is stained using SYBR DNA-stain at top, and using Coomassie Brilliant Blue (CBB) at bottom. RNF168 concentrations used are 50 µM, 25 µM, 12.5 µM, 6.25 µM, 3.13 µM, and 1.56 µM. (B) The R63A mutant shows a markedly reduced in affinity compared to wild-type as judged from the disappearance of the nucleosome band (labeled NCP) upon increasing RNF168 protein concentration. In addition, a protein-DNA complex at relatively high mobility is formed (labeled with *) indicating an altered binding mode. (C) Judging from the disappearance of the nucleosome band (labeled NCP) upon increasing RNF168 protein concentration, the R67A/R68A mutant protein has comparable affinity as wild-type for nucleosome binding. The absence of a well-defined band as for the wild-type suggest decreased stability of the complex and/or an altered binding mode.

A

	# / crosslink	SASD		# / crosslink	SASD
**Total reproducible crosslinked peptides**	65			H2A-K36 / H2B-K82	11.5
**Total unqiue reproducible crosslinks**	62			H2A-K36 / H4-K77	29.0
**Crosslinks involving tails**	40			H2A-K36 / H4-K91	24.2
**Crosslinks within/between tails**	16			H2A-K95 / H2B-K105	22.0
**Crosslinks within histone core**	21			H2A-K95 / H4-K91	21.5
**intramolecular**	6			H2B-K31 / H2B-K82	14.6
	H2A-K95 / H2A-K118	40.9*		H2B-K31 / H4-K91	24.3
	H2A-K95 / H2A-K119	42.4*		H3-K56 / H2A-K74	18.6
	H2B-K31 / H2B-K43	31.1		H3-K56 / H4-K91	49.5*
	H3-K56 / H3-K64	26.9		H3-K79 / H2B-K105	17.0
	H3-K56 / H3-K79	55.8*		H3-K79 / H4-K77	12.3
	H4-K77 / H4-K79	8.6		H3-K79 / H4-K79	9.9
**intermolecular**	15			H4-K91 / H2B-K82	23.6
	H2A-K36 / H2A-K36	17.7	**Intermolecular crosslinks RNF168-histones**	1	
	H2A-K36 / H2B-K31	17.2		H2B-K105 / RNF168-K46	8.7 ± 1.1

B

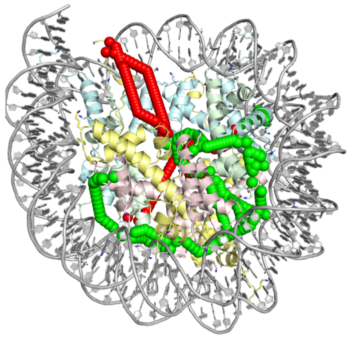

C

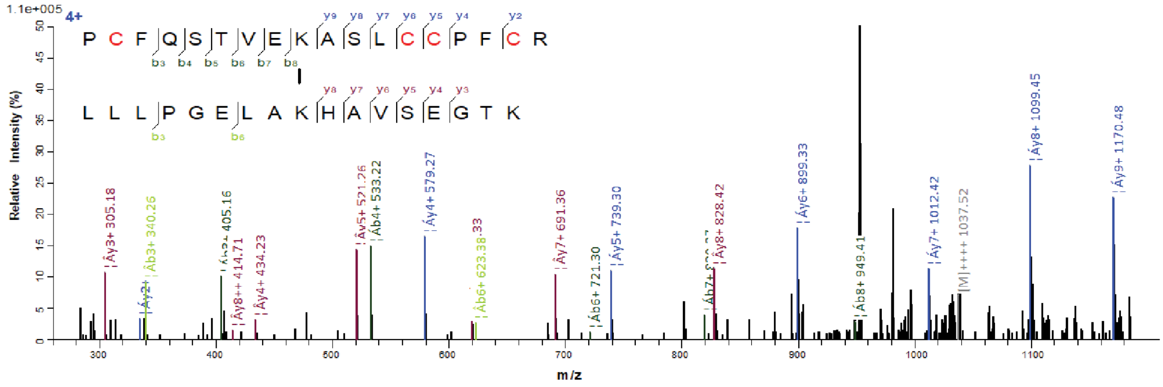

Supplementary Figure 3. Crosslinking mass-spectrometry results for the RNF168^{RING}–nucleosome complex. (A) Overview of all crosslinks obtained that were reproduced over three replicate experiments. The number of crosslinks obtained in various categories are indicated, as well as the identity of crosslinks that do not involve lysines in the flexible histone tails. For each listed crosslink, the solvent accessible surface distance (SASD) between Cα-atoms was calculated using Jwalk[1] based on the nucleosome crystal structure (1KX5). For the intermolecular crosslinks between RNF168^{RING} and the nucleosome, the SASD represents the average distance and standard deviation in the 10 best scoring structures of cluster 1. Out of 21 histone-histone crosslinks, 17 have SASD values that are compatible with cross-linking, while four have SASDs more than 40 Å (marked with *). For two of these, the cross-link involved lysines in the H2A C-terminal tail (K118/K119) which likely experiences dynamics and can thus transiently visit conformations that bring it close to H2A K95. The other two incompatible cross-links involve H3K56. These may be the result of transiently captured nucleosome-nucleosome contacts. (B) The solvent accessible surface distance (SASD) for the crosslinks involving the histone core are shown as spheres on the nucleosome structure. Incompatible crosslinks are shown in red, otherwise in green. (C) Mass spectrum and fragmentation pattern for the intermolecular crosslinked peptide as interpreted by the pLink software[2]. The peptide sequence is shown in the top, the top part of the sequence refers to RNF168, the bottom part to H2B. Crosslinking data is deposited in PRIDE database, accession code PXD012723.

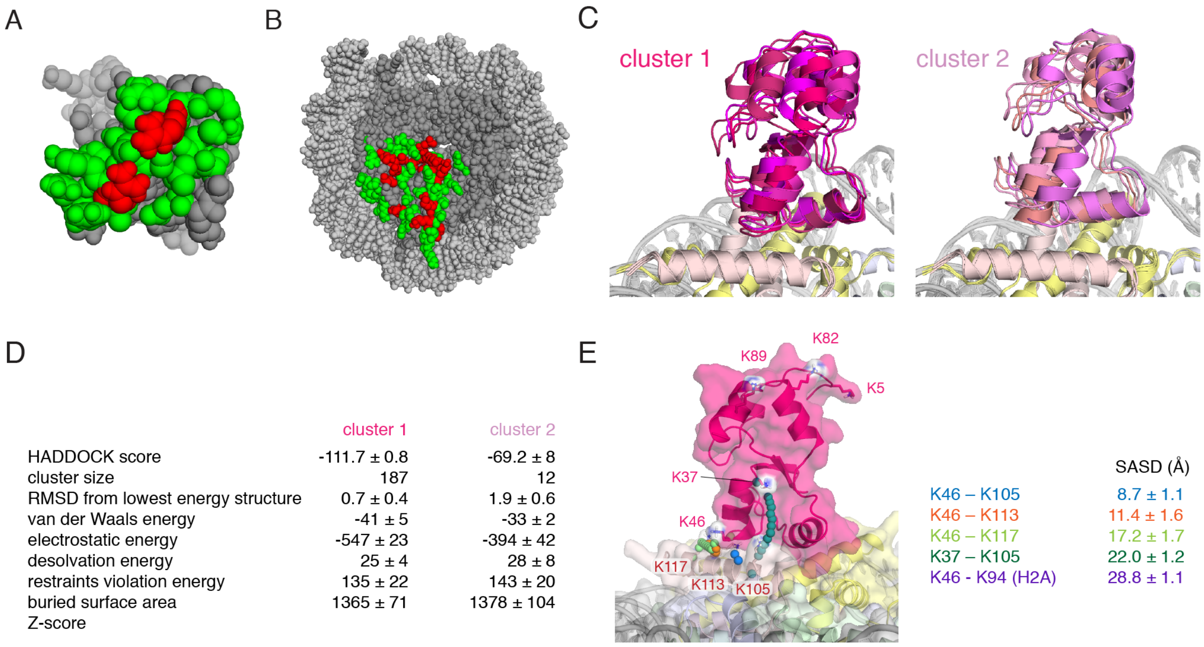

Supplementary Figure 4. Overview of the docking solutions for the RNF168^{RING}-nucleosome complex. (A,B) Definition of active and passive residues as input for the HADDOCK docking protocol. Views on the mapped binding interfaces of RNF168 (A, PDB-4GB0) and the nucleosome (B, PDB 2PYO) from mutagenesis and NMR data. Active residues are shown in red, passive residues in green and all others in gray. DNA is shown in light gray. (C,D) Superposition of (C) and statistics for (D) the best four structures of each cluster obtained by NMR, XL-MS and mutagenesis data driven docking of RNF168^{RING} to the nucleosome. Cluster 2 is a slightly tilted solution compared to cluster 1 and would position the E2 active site at ~13 Å from the target lysine. Error bars are s.d. over the four best structures. (E) Overview of all expected intermolecular crosslinks between RNF168^{RING} and the nucleosome based on the best ten solutions in cluster 1. Only crosslinks with solvent accessible surface distance (SASD) < 30 Å in all solutions were considered. All crosslinks are to lysines of H2B unless noted otherwise. Of the five expected crosslinks, four involve RNF168 K46. The experimentally observed crosslink also involves K46 and has the shortest predicted SASD. The SASD of K46-K94 (H2A) partially overlaps with the SASD path of the experimentally observed crosslink and is not shown for clarity. Error bars are s.d. over the best ten structures.

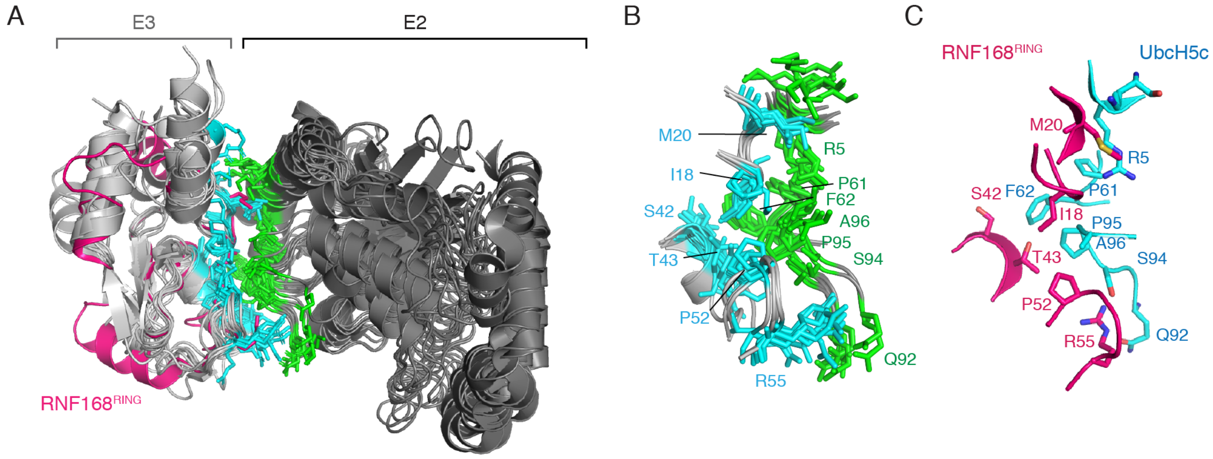

Supplementary Figure 5. Modelling of the E2-E3 interface. (A) Superposition of twelve E2-E3 structures with either high structural or sequence similarity to RNF168^{RING}. The interaction surfaces are color coded in blue for the E3 and green for the E2. Structures were superimposed on the E3. (B) Zoom on the intermolecular E2-E3 interface for the UbcH5 family, with conserved interactions shown in stick representation. Contacts between the labeled residues were imposed during docking of the ternary UbcH5c-RNF168^{RING}-nucleosome complex. In addition, the 'linchpin' hydrogen bond characteristic of active E3-E2~Ub complexes[3] was enforced between RNF168 R55 and UbcH5c Q92. (C) Zoom on the intermolecular E2-E3 interface in the best scoring solution for the UbcH5c-RNF168^{RING}-nucleosome complex.

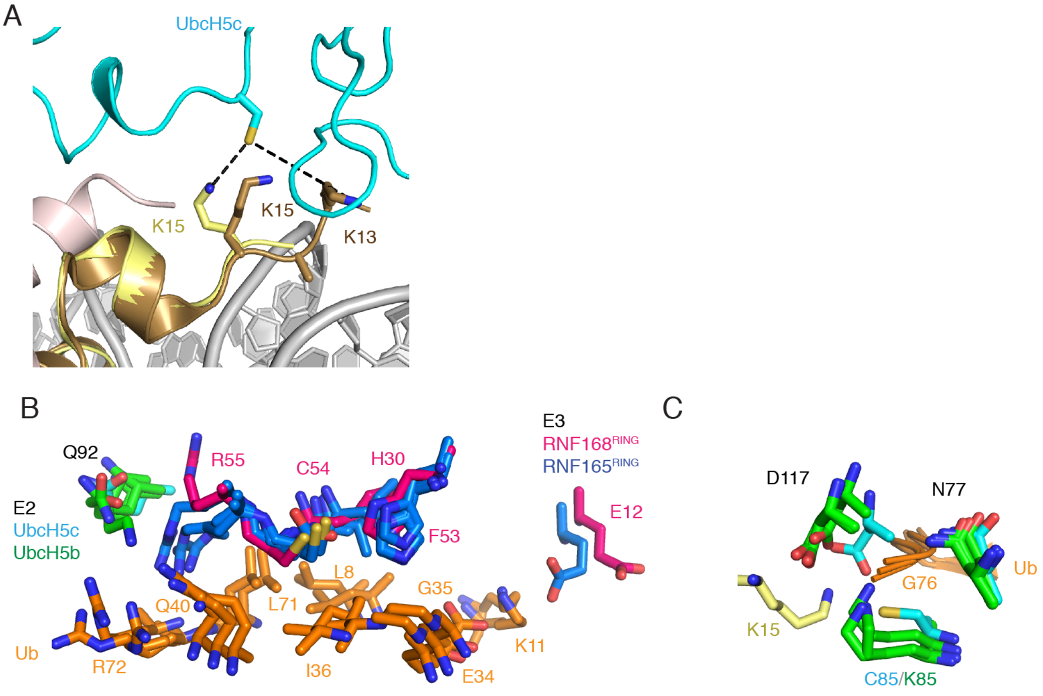

Supplementary Figure 6: Implications for Ub-transfer. (A) Superposition of *Hs.* nucleosome structure (PDB-ID 2CV5) with the RNF168-UbcH5c-nucleosome model. Side chains of H2A K13 and K15 are shown as brown sticks. In our model the Sγ-Nζ distance is 3.3 Å for K15. The distance to K13 is 10.8 Å, suggesting that ubiquitination of this residues involves a conformational change in this region of the dynamic H2A tail. (B) Detailed view on the conserved E3-Ub interface, including a potential salt-bridge or hydrogen interaction between Ub-K11 and RNF168-E12, a hydrogen bond between backbone of Ub-G35 and RNF168-H30, and a hydrophobic interaction between Ub residues L71, L8, I36 with RNF168 residues C54, and F53. (C) Detailed view on the catalytic center in the closed-state E3-E2~Ub structures superposed on the E3-E2-nucleosome model for RNF168. Crucial side chains are properly aligned to enable Ub transfer, further validating our model. D117, which has been termed the gateway residue, is hydrogen-bonding to the target lysine in our model. This may promote activation of the target lysine by stabilizing its deprotonation. Structures in panels (A) are superimposed on the RING domain of the E3, in panel (B) on the E2. Color coding indicated in the Figure.

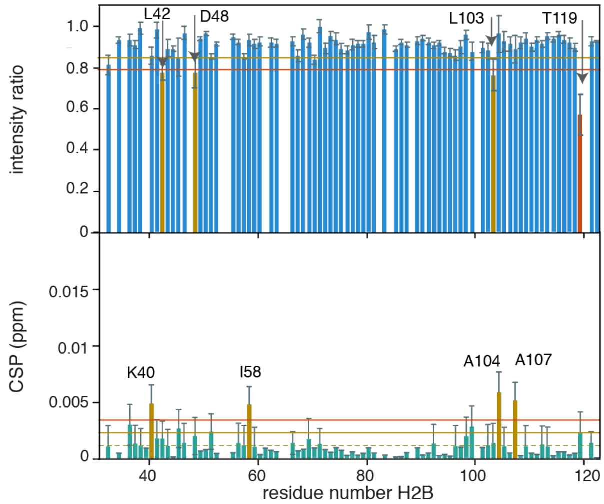

Supplementary Figure 7: Test of E2 histone-dimer interaction. Normalized intensity ratios (top) and chemical shift perturbations (CSPs, bottom) observed for H2B within H2A/H2B dimers upon addition of 0.05 molar equivalents Ubch5c in the presence of 0.16 equivalents of RNF168. Residues with CSPs that are one (two) standard deviation higher, or with intensities one (two) s.d. lower than the one-sided 10% trimmed mean, are color coded yellow (red) and labeled. The small effects are observed for the same set of residues as for RNF168 addition (see main text Figure 1 and Supplementary Figure 1). This suggest the ternary complex is not stable enough to be observed under these conditions. Addition of more equivalents of E2 resulted in problems with complex stability. Error bars are s.d. based on 1/10 ppb standard errors in $^{1}H/^{15}N$ peak position for the bottom and on noise levels for the top panel .

Supplementary references

1. Matthew Allen Bullock, J., Schwab, J., Thalassinos, K. & Topf, M. The Importance of Non-accessible Crosslinks and Solvent Accessible Surface Distance in Modeling Proteins with Restraints From Crosslinking Mass Spectrometry. *Mol Cell Proteomics* **15,** 2491–2500 (2016).
2. Yang, B. *et al.* Identification of cross-linked peptides from complex samples. *Nat Methods* **9,** 904–906 (2012).
3. Pruneda, J. N. *et al.* Structure of an E3:E2~Ub complex reveals an allosteric mechanism shared among RING/U-box ligases. *Mol. Cell* **47,** 933–942 (2012).
