# Supplementary material for: Structural basis of specific H2A K13/K15 ubiquitination by RNF168
Source: Nat Commun. 2019 Apr 15;10:1751. doi: 10.1038/s41467-019-09756-z (PMC6465349; doi:10.1038/s41467-019-09756-z)
Supplement: Supplementary file 3 — Reporting Summary [file 41467_2019_9756_MOESM3_ESM.pdf]

nature research

Corresponding author(s): van Ingen

Last updated by author(s): Feb 20, 2019

Reporting Summary

Nature Research wishes to improve the reproducibility of the work that we publish. This form provides structure for consistency and transparency in reporting. For further information on Nature Research policies, see Authors & Referees and the Editorial Policy Checklist.

Statistics

For all statistical analyses, confirm that the following items are present in the figure legend, table legend, main text, or Methods section.

n/a	Confirmed	
☒	☐	The exact sample size (*n*) for each experimental group/condition, given as a discrete number and unit of measurement
☒	☐	A statement on whether measurements were taken from distinct samples or whether the same sample was measured repeatedly
☒	☐	The statistical test(s) used AND whether they are one- or two-sided<br>*Only common tests should be described solely by name; describe more complex techniques in the Methods section.*
☒	☐	A description of all covariates tested
☒	☐	A description of any assumptions or corrections, such as tests of normality and adjustment for multiple comparisons
☒	☐	A full description of the statistical parameters including central tendency (e.g. means) or other basic estimates (e.g. regression coefficient) AND variation (e.g. standard deviation) or associated estimates of uncertainty (e.g. confidence intervals)
☒	☐	For null hypothesis testing, the test statistic (e.g. *F*, *t*, *r*) with confidence intervals, effect sizes, degrees of freedom and *P* value noted<br>*Give P values as exact values whenever suitable.*
☒	☐	For Bayesian analysis, information on the choice of priors and Markov chain Monte Carlo settings
☒	☐	For hierarchical and complex designs, identification of the appropriate level for tests and full reporting of outcomes
☒	☐	Estimates of effect sizes (e.g. Cohen's *d*, Pearson's *r*), indicating how they were calculated

Our web collection on statistics for biologists contains articles on many of the points above.

Software and code

Policy information about availability of computer code

Data collection	NMR: TopSpin 2.1 & 3.2
Data analysis	NMR: TopSpin 2.1 & 3.2, NMRPipe 9.8, NMRFAM-SPARKY 1.414<br>MS: pLink 2.3.4<br>modeling: HADDOCK 2.2

For manuscripts utilizing custom algorithms or software that are central to the research but not yet described in published literature, software must be made available to editors/reviewers. We strongly encourage code deposition in a community repository (e.g. GitHub). See the Nature Research guidelines for submitting code & software for further information.

Data

Policy information about availability of data

All manuscripts must include a data availability statement. This statement should provide the following information, where applicable:

- Accession codes, unique identifiers, or web links for publicly available datasets
- A list of figures that have associated raw data
- A description of any restrictions on data availability

NMR data that support the findings of this study have been deposited in the BMRB with the accession codes 27547 for the backbone assignment of H2A in the H2A/H2B dimer, 27791 and 27792 for the titration using histone H2A- or H2B-labeled H2A/H2B dimers and 27786 for the titration using H2A- and H2B-labeled nucleosomes (http://www.brmrb.wisc.edu.gov). Proteomics cross-linking data are available via ProteomeXchange under accession code PXD012723 (http://www.ebi.ac.uk/pride/archive). Structural models for RNF168-RING-nucleosome complex and the UbcH5c-RING-nucleosome complex are in the PDB-Dev database with the accession codes PDBDEV00000028 and PDBDEV00000029 (http://pdb-dev.wwpdb.org). All other data that support the findings of this study are available from the corresponding author upon reasonable request. The source data underlying Figs. 1–4 and Supplementary Figures 1, 2 and 4 are provided as a Source Data file.

Field-specific reporting

Please select the one below that is the best fit for your research. If you are not sure, read the appropriate sections before making your selection.

☒ Life sciences ☐ Behavioural & social sciences ☐ Ecological, evolutionary & environmental sciences

For a reference copy of the document with all sections, see nature.com/documents/nr-reporting-summary-flat.pdf

Life sciences study design

All studies must disclose on these points even when the disclosure is negative.

Sample size	NMR experiments are performed on single samples .<br>XL-MS was performed on 3 seperately prepared samples of the RNF168-nucleosome complex, with proteins from the same batch.<br>Ubiquitination and EMSA were performed on single samples
Data exclusions	No data was excluded other than in these two pre-established cases:<br>- residues with significant CSPs or intensity changes were excluded from the list of active residues in HADDOCK modeling if they had less than 25% side chain solvent exposure in the nucleosome<br>- crosslinks were excluded from consideration if they were not reproduced in three replicate experiments
Replication	NMR data were performed on 3 different samples (H2A/H2B dimer with H2A labeled, H2A/H2B dimer with H2B labeled, nucleosome with H2A/H2B labeled)<br>Ubiquitination assays and EMSA were generally performed in 2 replicates, with one shown in Figures<br>XL-MS assay was performed in 3 replicates
Randomization	n/a
Blinding	n/a

Reporting for specific materials, systems and methods

We require information from authors about some types of materials, experimental systems and methods used in many studies. Here, indicate whether each material, system or method listed is relevant to your study. If you are not sure if a list item applies to your research, read the appropriate section before selecting a response.

Materials & experimental systems

n/a	Involved in the study	
☒	☐	Antibodies
☒	☐	Eukaryotic cell lines
☒	☐	Palaeontology
☒	☐	Animals and other organisms
☒	☐	Human research participants
☒	☐	Clinical data

Methods

n/a	Involved in the study	
☒	☐	ChIP-seq
☒	☐	Flow cytometry
☒	☐	MRI-based neuroimaging
