# Supplementary material for: Structural basis of specific H2A K13/K15 ubiquitination by RNF168
Source: Nat Commun. 2019 Apr 15;10:1751. doi: 10.1038/s41467-019-09756-z (PMC6465349; doi:10.1038/s41467-019-09756-z)
Supplement: Supplementary file 4 — Source Data [file 41467_2019_9756_MOESM4_ESM.zip › SourceData.tar!/SOURCE-DATA-HORN-ETAL/FigureS2/FigureS2-panelA-1H-1D-RNF168-WT.pdf]

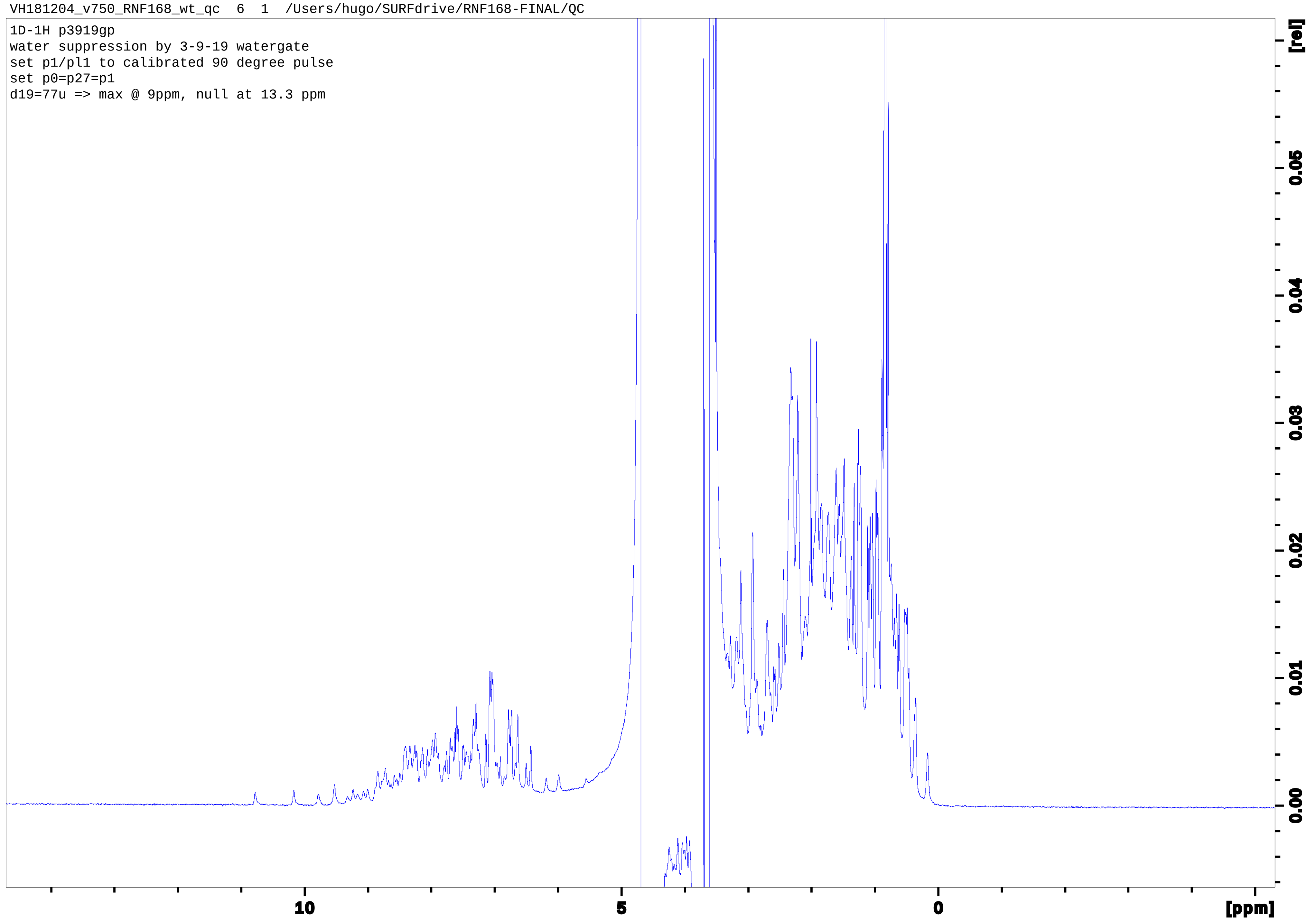

VH181204_v750_RNF168_wt_qc 6 1 /Users/hugo/SURFdrive/RNF168-FINAL/QC
1D-1H p3919gp
water suppression by 3-9-19 watergate
set p1/pl1 to calibrated 90 degree pulse
set p0=p27=p1
d19=77u => max @ 9ppm, null at 13.3 ppm
[rel]
0.05
0.04
0.03
0.02
0.01
0.00
10
5
0
[ppm]
