# Supplementary material for: Structural basis of specific H2A K13/K15 ubiquitination by RNF168
Source: Nat Commun. 2019 Apr 15;10:1751. doi: 10.1038/s41467-019-09756-z (PMC6465349; doi:10.1038/s41467-019-09756-z)

VH181212_v750_RNF168_R63A_qc 10 1 /Users/hugo/SURFdrive/RNF168-FINAL/QC

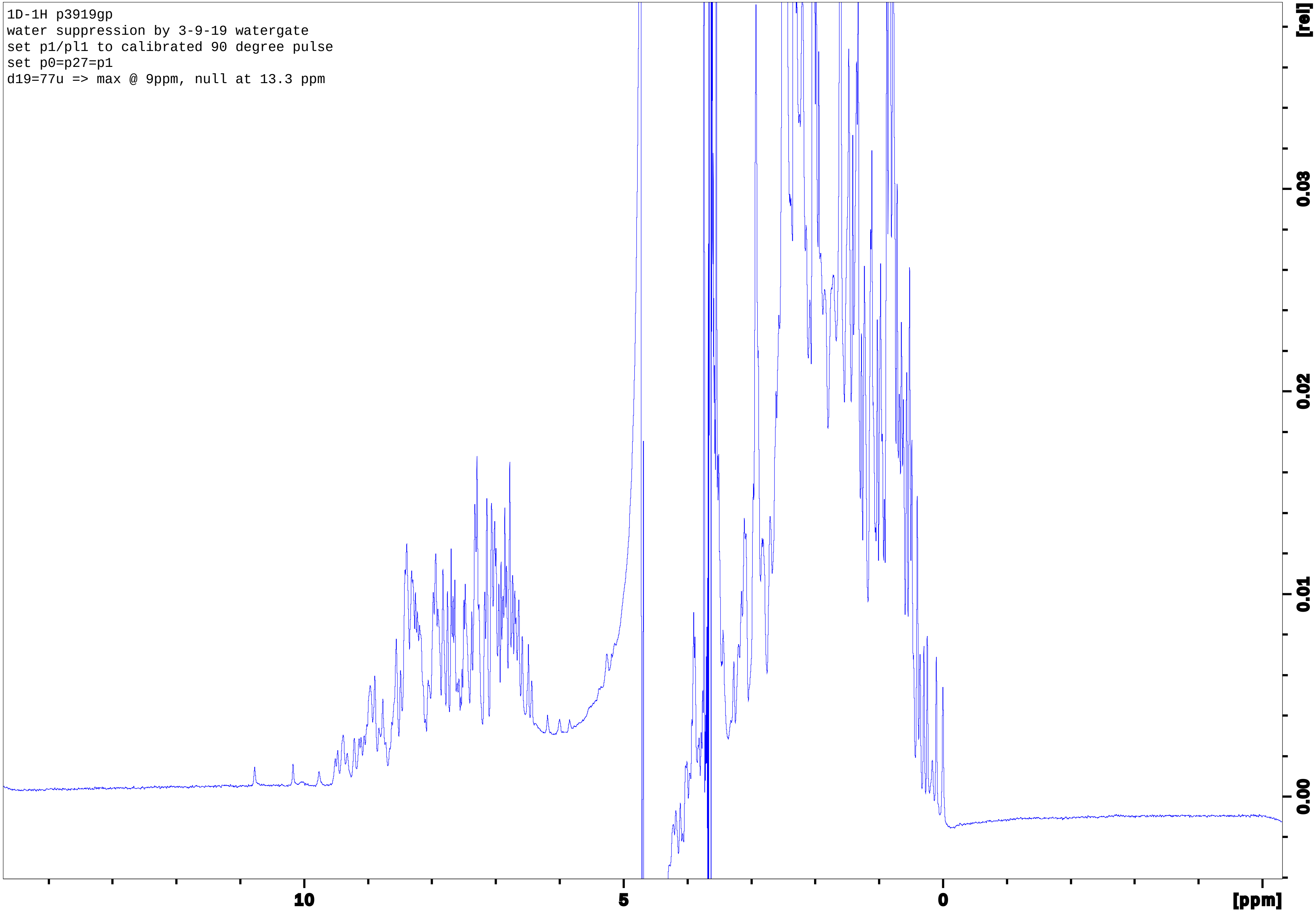

Supplement: Supplementary file 4 — Source Data [file 41467_2019_9756_MOESM4_ESM.zip › SourceData.tar!/SOURCE-DATA-HORN-ETAL/FigureS2/FigureS2-panelA-1H-1D-RNF168-R63A.pdf]
